# Draft Genome Sequences of *Serratia marcescens* Strains CAPREx SY13 and CAPREx SY21 Isolated from Yams

**DOI:** 10.1128/genomeA.00191-17

**Published:** 2017-05-11

**Authors:** Joseph Honger, Rita E. Monson, Alison Rawlinson, George P. C. Salmond

**Affiliations:** Department of Biochemistry, University of Cambridge, Cambridge, United Kingdom

## Abstract

*Serratia marcescens* strains CAPREx SY13 and CAPREx SY21 were isolated from Ghanaian yams from a London market. The draft genomes suggest that the strains are similar, with genomes of 5,308,004 and 5,157,134 bp and 59.35 and 59.62 G+C%, respectively. The genes necessary for prodigiosin biosynthesis were present in both strains.

## GENOME ANNOUNCEMENT

*Serratia marcescens* is a Gram-negative rod-shaped enterobacterium and a frequent cause of hospital acquired infections. However, *S. marcescens* is also known to reside in the soil and has been reported to infect agricultural crops such as squash (1, 2). Clinical isolates of* S. marcescens* often show resistance to antibiotics and are known to produce β-lactamases (2, 3). *S. marcescens* strains often produce the red pigment, 2-methyl-3-pentyl-6-methoxyprodiginine (prodigiosin) (4). Here, we report the genomes of two *S. marcescens* strains CAPREx SY13 and CAPREx SY21, isolated from Ghanaian yams from a London market. The two strains were able to grow at 30°C and 37°C and were selected for further analysis due to their red pigmentation.

Both strains were sequenced using Illumina HiSeq using 2 × 250 bp paired-end reads. Reads were trimmed using Trimmomatic (5) and assembled using SPAdes (6). Prokka (7) was used to generate an initial annotation of the genome and PGAP was used to annotate the final versions in GenBank. The quality of the assembly and sequencing was analyzed using BWA-mem (8). The draft genome of CAPREx SY13 was 5,308,004 bp with 59.35 G+C% and 90 tRNAs and a mean coverage of 79.32×. CAPREx SY21 was 5,157,134 bp with 59.62 G+C%, 95 tRNAs and a mean coverage of 82.6×.

We were able to identify several potential secondary metabolite clusters in both CAPREx SY13 and CAPREx SY21. Both strains encode a cluster similar to the biosynthetic cluster of prodigiosin in *S. marcescens* ATCC 274 (9). The genomic context of the prodigiosin biosynthetic cluster, flanked by *copA* and *cueR*, was conserved in all three strains. The two strains were also predicted to encode machinery for the production of a surfactant similar to serrawettin. Many clinical isolates of *S. marcescens* are resistant to β-lactam antibiotics (2, 3, 10). Both CAPREx SY13 and CAPREx SY21 encode a single β-lactamase of the AmpC family and another putative metallo-β-lactamase. Both strains also encode chitinases, again characteristic of clinical *S. marcescens* isolates (2, 11, 12).

We were unable to identify an N-acyl-homoserine lactone quorum-sensing system in either strain, though both strains did possess a *luxS* orthologue. CAPREx SY13 and CAPREx SY21 each encode a lactonase-like protein similar to YtnP, a protein from *Bacillus subtilis* that affects streptomycin production and aerial mycelium development in *Streptomyces griseus* (13). Both strains carry the genes predicted to encode QseGEF, a system that allows sensing and response to epinephrine, phosphate, and sulfate in enterohemorrhagic *Escherichia coli* (14).

These draft genomes contain many genes characteristic of *S. marcescens*, including the prodigiosin biosynthetic cluster. Both strains also encode antibiotic resistance genes, common to some clinical *S. marcescens* isolates, despite being isolated from yams. We hope that these two genomes will help further our understanding of *S. marcescens* physiology in soil and plant niches.

### Accession number(s).

The draft genomes of these strains have been deposited in GenBank under accession numbers MVGA00000000 (SY13) and MVGB00000000 (SY21).
